# Glucagon-Like Peptide-1 (GLP-1) and 5-Hydroxytryptamine 2c (5-HT_2c_) Receptor Agonists in the Ventral Tegmental Area (VTA) Inhibit Ghrelin-Stimulated Appetitive Reward

**DOI:** 10.3390/ijms20040889

**Published:** 2019-02-19

**Authors:** Erin Howell, Hannah M. Baumgartner, Lia J. Zallar, Joaquín A. Selva, Liv Engel, Paul J. Currie

**Affiliations:** Department of Psychology, Reed College, 3203 SE Woodstock Blvd., Portland, OR 97202, USA; howelle@reed.edu (E.H.); hmbaum@umich.edu (H.M.B.); lia.zallar@gmail.com (L.J.Z.); jselva@alumni.reed.edu (J.A.S.); engelol@reed.edu (L.E.)

**Keywords:** appetitive reward, operant responding, mesolimbic, Ex-4, Ro60-0175

## Abstract

Current literature indicates that the orexigenic peptide ghrelin increases appetitive motivation via signaling in the mesolimbic reward system. Another gastric peptide, glucagon-like peptide-1 (GLP-1), and the neurotransmitter 5-hydroxytryptamine (5-HT), are both known to suppress operant responding for food by acting on key mesolimbic nuclei, including the ventral tegmental area (VTA). In order to investigate the interaction effects of ghrelin, GLP-1, and 5-HT within the VTA, we measured operant responding for sucrose pellets after the administration of ghrelin, the GLP-1 receptor agonist exendin-4 (Ex-4), and the 5-HT_2c_ receptor agonist Ro60-0175 in male Sprague-Dawley rats. Following training on a progressive ratio 3 (PR3) schedule, animals were first injected with ghrelin into the VTA at doses of 3 to 300 pmol. In subsequent testing, separate rats were administered intraperitoneal (IP) Ex-4 (0.1–1.0 µg/kg) or VTA Ex-4 (0.01–0.1 µg) paired with 300 pmol ghrelin. In a final group of rats, the 5-HT_2c_ agonist Ro60-0175 was injected IP (0.25–1.0 mg/kg) or into the VTA (1.5–3.0 µg), and under both conditions paired with 300 pmol ghrelin delivered into the VTA. Our results indicated that ghrelin administration increased operant responding for food reward and that this effect was attenuated by IP and VTA Ex-4 pretreatment as well as pre-administration of IP or VTA Ro60-0175. These data provide compelling evidence that mesolimbic GLP-1 and serotonergic circuitry interact with the ghrelinergic system to suppress ghrelin’s effects on the mediation of food reinforcement.

## 1. Introduction

The 28-amino acid peptide ghrelin is known to stimulate food intake and metabolic responding by binding growth hormone secretagogue 1a receptors (GHS-R1a) in the hypothalamus [1,2,3]. Initially identified by Kojima et al. in 1999 [4], ghrelin is predominantly synthesized in the gut, but crosses the blood–brain barrier to activate receptors in regions of the central nervous system (CNS) [5]. In addition to increasing appetite, the peptide stimulates incentive motivation for food by action in the mesolimbic reward system [6,7]. Ghrelin binding to the GHS-R1a expressed in the hypothalamus, as well as the ventral tegmental area (VTA) and other regions of the mesolimbic dopamine circuit, mediates the peptide’s effects on behavior [5,8]. Within the VTA, ghrelin increases incentive motivation for food [9,10] and contributes to elevated dopamine release in the nucleus accumbens (NAcc) [5]. Ghrelin also reportedly impacts mesolimbic activity by stimulating cholinergic projections from the laterodorsal tegmental area (LDTg) to the VTA [11]. Growing evidence suggests that ghrelin increases drug reward as well [2,12,13,14]. Administration of the peptide into the VTA potentiates cocaine-induced conditioned place preference [14] and increases alcohol intake [2]. Ghrelin receptor deletion significantly reduces alcohol intake [13], and alcohol consumption reduces VTA ghrelin receptor expression [8]. The peptide therefore represents an important regulator of rewarding experience and is implicated in the treatment of obesity and addiction. 

Past reports suggest that ghrelin interacts with the peptide GLP-1 and the monoamine transmitter 5-hydroxytryptamine (5-HT) in the regulation of food intake and reward [15,16]. GLP-1 is a gastrically-derived anorexigenic peptide produced from intestinal L-cells [17]. Secretion of GLP-1 is triggered by nutrient availability in the stomach, after which the peptide is transported to the liver before entering systemic circulation [17]. Additionally, in the CNS, GLP-1 is secreted by the nucleus tractus solitarius (NTS) of the hindbrain [18]. Receptor expression analysis indicates that GLP-1 receptors are highly expressed in the hypothalamus but also localize to neurons in the VTA, NAcc, and 5-HT producing raphe neurons [19]. Notably, NTS GLP-1-producing neurons project directly to the VTA and the NAcc, and stimulation of these projections inhibits food intake and contributes to body weight reduction [18]. In the hypothalamus, GLP-1 receptor activation reduces food intake and energy metabolism and attenuates ghrelinergic increases in food intake and metabolism [1,15]. GLP-1 receptor activation also affects reward-related behaviors such as alcohol intake and alcohol-mediated conditioned place preference [20], amphetamine-induced conditioned place preference, and hedonic feeding [21], as well as cocaine self-administration [22]. These effects were all observed following injection of the GLP-1 receptor agonist exendin-4 (Ex-4). On the other hand, 5-HT is recognized as an important mesolimbic transmitter, particularly within the VTA [23,24]. Fletcher et al. reported that intra-VTA administration of the 5-HT_2C_ receptor agonist Ro60-0175 inhibits the locomotor effects of cocaine and reduces cocaine self-administration [24]. In fact, numerous reports have confirmed that 5-HT_2C_ activation reduces food intake and reward [25,26]. Notably, stimulation of mesolimbic 5-HT_2C_ receptors has potent effects on incentive motivation [23,27]. These receptors are known to localize to VTA neurons to stimulate dopamine release in the NAcc [28]. One recent study found that 5-HT projections from the raphe to the VTA, but not to the NAcc, inhibit operant responding [29]. Given the effects of ghrelin, GLP-1, and 5-HT in both food and drug reward, the present report sought to investigate the interaction of their circuitry in appetitive motivation within the VTA. Specifically, operant responding was measured in rodents administered either Ex-4 or Ro60-0175 prior to VTA ghrelin treatment. 

## 2. Results

**Figure 1:**
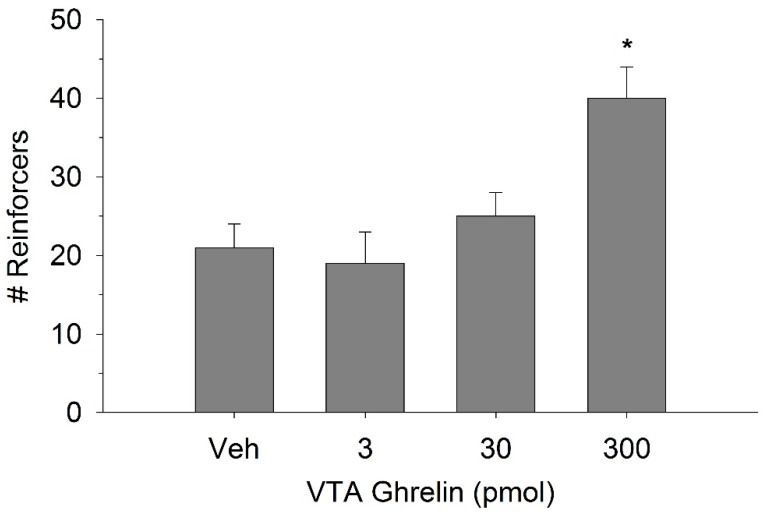
Ghrelin administration into the ventral tegmental area (VTA) increased operant responding for food reward. Values represent mean number of reinforcers (sucrose pellets) ± SEM; *n* = 8. * *p* < 0.05 compared to vehicle (Veh).

**Figure 2:**
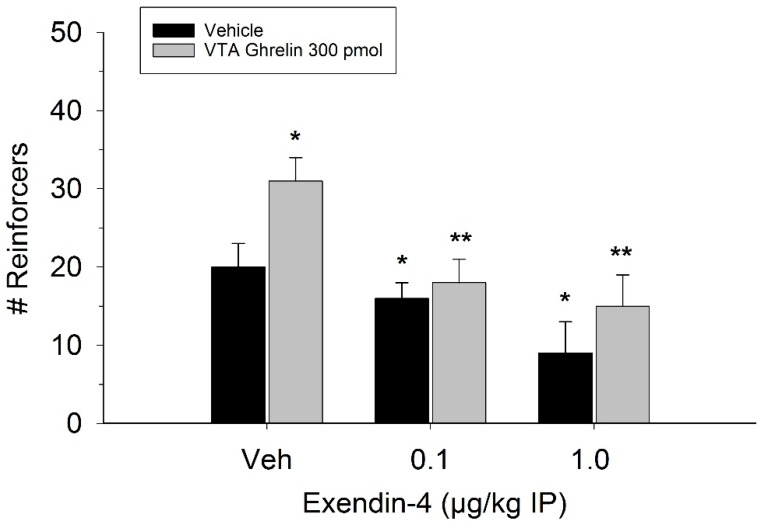
Peripheral injection of Ex-4 attenuated the effect of VTA ghrelin on operant responding. Values represent mean number of reinforcers ± SEM; *n* = 8. * *p* < 0.05 compared to vehicle (Veh). ** *p* < 0.05 compared to ghrelin paired with saline Veh pretreatment.

**Figure 3:**
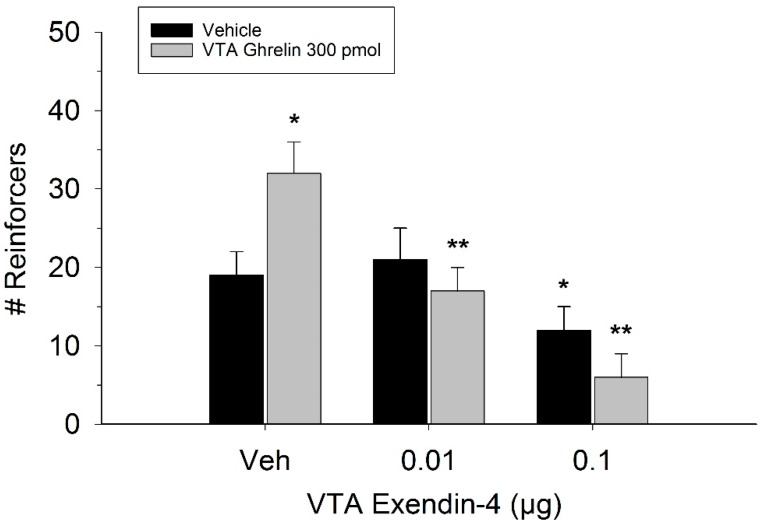
Effect of VTA Ex-4 pretreatment on ghrelin-induced operant responding for food. While only the higher dose of Ex-4 reduced responding when injected into the VTA alone, both doses of the GLP-1 agonist reliably attenuated ghrelin’s stimulatory action on food reinforcement. Values represent mean number of reinforcers ± SEM; *n* = 8. * *p* < 0.05 compared to vehicle (Veh). ** *p*< 0.05 compared to ghrelin administered with Veh pretreatment.

**Figure 4:**
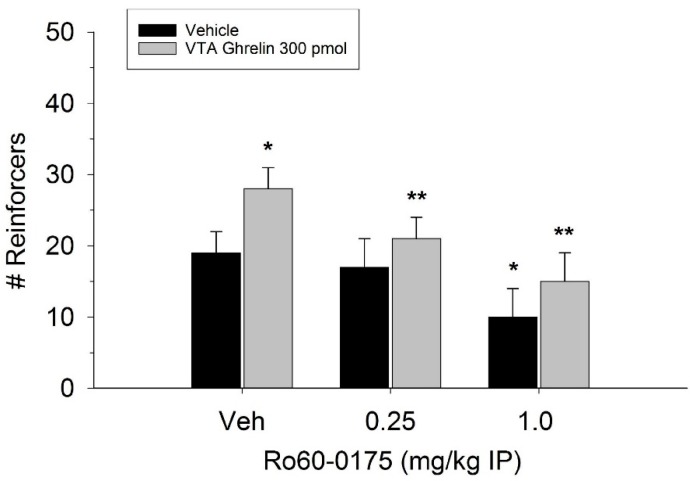
Systemic administration of the 5-HT_2c_ agonist Ro60-0175 attenuated the effect of VTA ghrelin on operant responding for food reward. Data are presented as mean number of reinforcers ± SEM; *n* = 8. * *p* < 0.05 compared to vehicle (Veh). ** *p* < 0.05 compared to ghrelin administered with saline pretreatment.

**Figure 5:**
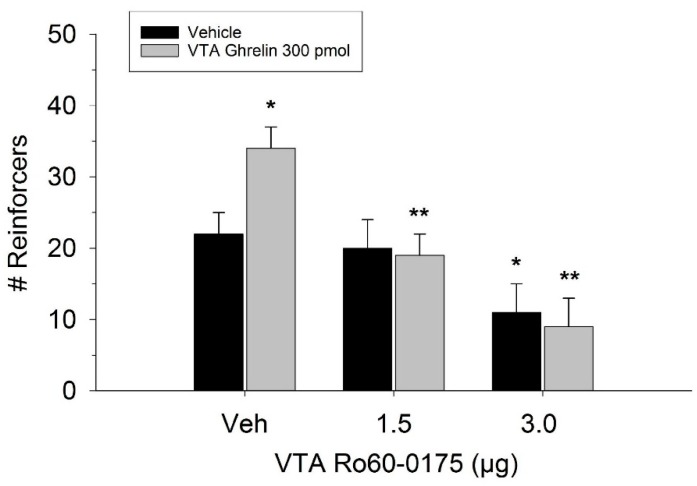
Effect of Ro60-0175 and ghrelin co-administration into the VTA on operant responding for food reward. Both doses of Ro60-0175 inhibited the effect of ghrelin as observed with a significant reduction in the number of reinforcers acquired compared to the ghrelin/saline treatment condition. The higher dose of Ro60-0175 was also found to suppress responding when paired with vehicle (Veh). Data are represented as mean number of reinforcers ± SEM; *n* = 8. * *p* < 0.05 compared to Veh. ** *p* < 0.05 compared to ghrelin co-injected with saline.

The effect of ghrelin administration into the VTA is shown in Figure 1. One-way repeated measures ANOVA indicated that ghrelin, at a dose of 300 pmol, increased operant responding for food reward, reflected as an increase in the number of reinforcers acquired (F(3,21) = 27.4, *p* < 0.01). Figure 2 and Figure 3 demonstrate the effect of the GLP-1 agonist, Ex-4, on ghrelin-stimulated operant responding for food reward following systemic or direct VTA administration respectively. Data were analyzed by separate two-way (3 × 2; Ex-4 dose x ghrelin dose) repeated measures ANOVA. While IP (F(2,14) = 7.1, *p* < 0.01) and VTA (F(2,14) = 16.8, *p* < 0.001) Ex-4 suppressed responding, more importantly, both systemic and VTA Ex-4 reliably attenuated the stimulatory effect of ghrelin. It is noteworthy that the lower dose of 0.01 µg of VTA Ex-4 did not reliably alter responding on its own but this same dose effectively attenuated ghrelin-induced operant responding for food reinforcement. In separate rats we then investigated the effect of Ro60-0175 via a two-way (3 × 2; Ro60-0175 dose x ghrelin dose) repeated measures ANOVA to determine if 5-HT_2c_ agonism would elicit comparable effects to Ex-4. Whereas the highest dose of systemic and VTA Ro60-0175 decreased operant responding when paired with saline, all doses of Ro60-0715 significantly reduced responding elicited by ghrelin. That is, when injected IP (F(2,14) = 5.9, *p* < 0.01) or into the VTA (F(2,14) = 10.9, *p* < 0.001), Ro60-0175 pretreatment suppressed the stimulatory action of VTA ghrelin on operant responding for food reward (see Figure 4 and Figure 5).

## 3. Discussion

In accordance with previous research on ghrelinergic signaling within the mesolimbic reward system [6,7,9,10,15,30,31], we have demonstrated a significant increase in PR3 operant responding following VTA injection of 300-pmol ghrelin. Both IP and direct VTA administration of the GLP-1 receptor agonist Ex-4 significantly reduced operant responding as well as the increase in responding following ghrelin injection. Systemic and central administration of the 5-HT_2c_ receptor agonist Ro60-0175 also reliably reduced responding as well as the ghrelin induced increase in operant responding. Notably, Ro60-0175 and Ex-4 each attenuated ghrelin’s action even when injected at subthreshold doses. These findings suggest that GLP-1 and 5-HT systems crucially impact ghrelin’s effects on appetitive motivation within the VTA. Given that direct VTA administration of Ex-4 and Ro60-0175 attenuated ghrelin’s effects, it is reasonable to argue that systemic administration of both compounds acted within the VTA to inhibit ghrelin induced operant responding. 

Prior reports have shown that ghrelin receptor knockout reduces alcohol self-administration in an operant paradigm [13], supporting our finding that ghrelin affects incentive motivation. Additional studies indicate that reintroduction of GHSR-1a after knockout in the VTA increased food intake under stressful conditions and cocaine induced locomotor response [32]. Ghrelin receptor antagonism experiments revealed that inhibition of the receptor reduces food and drug motivation [12,33], suggesting that ghrelin may be necessary for functional reward processing. Specifically, VTA ghrelin and VTA GHSR-1a stimulation enhance operant responding for food [10,34] and increase cFos levels in a subset of VTA neurons [35]. The current investigation adds to previous findings of ghrelin’s effects on reward and provides novel evidence for an interaction of ghrelin with the anorexigenic peptide GLP-1 and monoaminergic transmitter 5-HT. 

With respect to the former, we found that systemic administration of Ex-4 at doses of 0.1 and 1.0 µg/kg reliably suppressed PR3 lever pressing for food reinforcement, indicating a reduction in food motivation. A report by Egecioglu et al. similarly found that 1.2 μg/kg IP Ex-4 reduced lever pressing on a progressive ratio schedule [20]. Additionally, prior research has assessed the effects of systemic GLP-1 receptor activation and inhibition on rewarding substances with no caloric value [21,22,36,37]. Hernandez et al. injected 3.0 μg/kg fluorescein-tagged Ex-4 IP and found reduced operant responding for cocaine with no effect on feeding behavior or body weight [22]. The authors further identified the fluorescein-tagged Ex-4 in VTA dopamine neurons [22]. In the same study, VTA administration of the GLP-1 receptor antagonist Exendin-(9-39) (Ex-9) attenuated the inhibitory effects of IP Ex-4 induced cocaine self-administration [22]. In addition to our own data, the above findings [22,36,37] support the hypothesis that peripherally-injected Ex-4 decreases incentive motivation by action in the VTA. 

GLP-1-producing neurons from the NTS are known to project to the VTA as well as the NAcc of the reward system [18]. The current report investigated direct VTA Ex-4 administration on operant responding for sucrose pellets and found a significant reduction in lever pressing on the PR3 schedule following the 0.1 μg dose. The lower dose of 0.01 μg Ex-4 did not significantly alter PR3 operant responding. Our findings indicating reduced responding after direct VTA Ex-4 administration provide evidence that the effects of systemic Ex-4 may result from the stimulation of VTA GLP-1 receptors. However, while Schmidt et al. found significant reduction in cocaine self-administration at a dose of 0.05 μg VTA Ex-4, they report no effect on responding for sucrose pellets at that dose [37]. Other work has identified significant effects of 0.05 μg VTA Ex-4 on food intake and body weight [18]. Therefore, more research is needed to determine the precise threshold of effect of VTA GLP-1 receptor activation on food reward.

In the present study, both systemic and VTA Ex-4 reliably attenuated the effect of 300 pmol ghrelin when compared to ghrelin paired with saline. Within the VTA, the lower dose of 0.01 μg Ex-4 significantly attenuated ghrelin’s effects despite having no effect when administered on its own. While current literature indicates that both peptides act within the mesolimbic reward system, to our knowledge no other study has identified an interaction of GLP-1 and ghrelin systems on reward within the VTA. Previous studies indicate that ghrelin stimulates gut GLP-1 release but inhibits GLP-1 transcription [38,39]. Other reports have found that central Ex-4 activates hypothalamic ghrelin neurons and reduces ghrelin mRNA [40]. Our lab has recently demonstrated that hypothalamic GLP-1 reduces ghrelinergic shifts in metabolism [1,6]. We have further shown that ghrelin and GLP-1 interact in the control of alcohol intake within the NAcc [6]. The present data therefore suggest that GLP-1 signaling regulates ghrelin’s impact on appetitive motivation within the VTA. 

The monoaminergic neurotransmitter 5-HT is also reported to be anorexigenic and to play an important role in regulating reward and incentive motivation, particularly within the VTA [24,25,26,27,29]. A recent investigation found a reduction in responding for food on both fixed ratio and progressive ratio operant schedules following IP injection of WAY163909, a 5-HT_2c_ agonist [25]. Another study similarly reported that the 5-HT_2c_ agonist lorcaserin suppressed binge food intake when administered IP [26]. In the current experiment, IP injection of the 5-HT_2c_ receptor agonist Ro60-0175 reliably reduced operant responding on a PR3 schedule at a dose of 1.0 mg/kg but not at a dose of 0.25 mg/kg. These data support findings from Fletcher et al. that 1.0 mg/kg but not 0.3 mg/kg subcutaneous Ro60-0175 injection decreases responding for both sucrose and chow rewards on a progressive ratio schedule [41]. Evidence from optogenetic research shows that 5-HT neuronal projections from the raphe to the VTA greatly affect incentive motivation [29]. In addition to our own data, these findings indicate that the reduction in operant responding produced by peripheral Ro60-0175 administration may be attributed, at least in part, to 5-HT_2C_ activation in the VTA. 

Therefore, we further investigated the role of mesolimbic 5-HT_2C_ receptors in the regulation of incentive motivation by administering Ro60-0175 directly into the VTA. While 3.0 μg VTA Ro60-0175 reliably reduced PR3 operant responding, the dose of 1.5 μg had no significant effect. Past studies have also demonstrated reduced motivation to self-administer cocaine or to respond for food on a progressive ratio schedule after 3 and 10 μg intra-VTA Ro60-0175 injection [24]. Xu et al. demonstrated that VTA 5-HT_2C_ receptor activation with the agonist lorcaserin prevents binge eating in mice [26]. Additional studies in mice reveal that 5-HT_2C_ agonism by peripheral lorcaserin injection increases cFos expression in VTA GABA neurons [27]. These data further indicate that the behavioral effects of systemic Ro60-0175 are mediated by VTA 5HT_2C_ receptor mechanisms. 

Similar to the interaction observed after ghrelin and GLP-1 coadministration, both systemic and VTA Ro60-0175 attenuated the effects of ghrelin on motivation to obtain food reinforcers, even at subthreshold doses. Previously, our lab has reported that within the hypothalamic paraventricular nucleus (PVN), 5-HT receptor activation reliably attenuates ghrelin’s effects on food intake and metabolism as measured by the respiratory exchange ratio [16]. The present results indicate that 5-HT may impact ghrelinergic signaling in the mesolimbic reward system in addition to the hypothalamus. Moreover, findings from Anderberg et al. indicate that disrupting 5-HT production reduced the effects of GLP-1 receptor activation on weight loss and that Ex-4 injection in the dorsal raphe reduced food intake [42], indicating there could be further interaction between ghrelin, GLP-1, and 5-HT systems. The mechanism by which ghrelin, GLP-1 and 5-HT each influence incentive motivation within VTA neurons remains to be determined. Ghrelin is believed to act in part via VTA dopaminergic neurons since 6-hydroxydopamine lesions of the VTA inhibit the peptide’s ability to elicit food reinforced behavior [43]. Recent evidence also indicates that ghrelin binds to GABAergic VTA neurons [35] and within the VTA GLP-1 receptor activation is believed to decrease dopamine release in the NAcc by interacting with dopamine, GABA, or glutamate neurons [22]. Though current literature indicates a strong role for VTA 5-HT in the control of incentive motivation, reports conflict as to whether 5-HT is acting directly on GABA [27] or dopamine neurons [24,26]. Bubar et al. present evidence that 5-HT_2C_ receptors are expressed on GABA and dopamine, as well as GABA and dopamine coexpressing neurons that project from the VTA to the NAcc [28]. Consequently, though ghrelin, GLP-1, and 5-HT clearly interact in the mesolimbic reward system, further work is needed to elucidate which neuronal populations are most affected by the neuropeptides and transmitter. 

The findings of the current study are in agreement with an emerging body of evidence implicating metabolic signals as modulators of reward and the reinforcing properties of drugs of abuse. In the hypothalamus, for example, these metabolic regulators of appetite, body weight, and energy metabolism, and/or their receptors, are expressed within distinct regions, including the arcuate nucleus. Here neuropeptide Y/agouti-related peptide (NPY/AgRP) neuronal activation is documented to increase food intake, promote weight gain, alter energy balance, and to increase energy substrate oxidation [1,44,45]. Interestingly, in vitro electrophysiological data confirm that ghrelin activates these same NPY/AgRP neurons while inhibiting the firing rate of proximal arcuate pro-opiomelanocortin (POMC) neurons [46,47,48]. This is consistent with ghrelin’s reported orexigenic and metabolic action via GHS-R1a [3,49]. In fact, POMC neurons produce contrasting effects on appetite and metabolism mediated, in part, via melanocortin, leptin, GLP-1, and 5-HT signaling [1,50,51,52,53]. Similar observations with respect to changes in food intake and energy metabolism have been reported in the PVN, a dorsal region of the hypothalamus, where, for example, 5-HT itself or direct 5-HT_2a/2c_ agonism inhibits alterations in eating and substrate oxidation elicited by NPY and ghrelin [16,54,55]. 

While the hypothalamic control of appetite necessarily represents complex interactions of multiple regulatory systems, what has become increasingly apparent is that many of these same neural and hormonal systems, as indicated above, impact mesolimbic reward. In addition to ghrelin, receptors for the orexigenic peptide NPY expressed in the VTA and NAcc are known to modify the rewarding properties of alcohol, opioids, and cocaine [56,57,58,59,60,61]. The NPY Y5 receptor colocolizes with VTA dopamine neurons [60], while the NPY Y1 receptor is expressed in the NAcc and antagonism of the receptor attenuates morphine-induced increases in operant responding for brain stimulation [57]. Ghrelin is also involved in the reinforcing properties of opioids. Inhibition of ghrelin receptors in the NAcc attenuates the effects of morphine [12] and fentanyl [62] on extracellular GABA and dopamine increase. In contrast to ghrelin and NPY, the anorexigenic peptide leptin decreases cocaine self-administration and attenuates DA neuronal firing in the VTA in response to food or cocaine [63,64,65]. Additionally, receptors for melanocortin are expressed on VTA dopamine neurons [66]. Stimulation of these receptors increases VTA dopamine neuronal activity as well as food reward [66], while blocking melanocortin 4 receptors (MC4), particularly in the NAcc, reduces cocaine reward [67]. 

In conclusion, the present report indicates that GLP-1 and 5-HT signaling crucially impact ghrelinergic behavioral response and appetitive motivation within the VTA. However, continued experimentation is needed to determine the precise mechanisms by which these molecules influence reinforcement as well as the effects of their interaction on binge eating, food palatability, and type of reinforcer. The current findings could inform the eventual development of treatments for addiction and obesity. 

## 4. Methods

### 4.1. Animals

Adult male Sprague-Dawley Rats (*n* = 40) were purchased from Envigo Laboratories (Madison, WI, USA) and were pair-housed upon arrival. At the time of surgery rats weighed 275–300 g and were moved to individual housing immediately following the cannulation procedure. All animals were maintained in a colony room on a 12 h light/dark cycle (lights out 1500 h) and room temperature was set at 22 ± 2 °C. Rats were given ad libitum access to standard rodent chow (LabDiet, St. Louis, MO, USA) and water. All experimental procedures were conducted during the nocturnal cycle and were approved by the Reed College Institutional Animal Care and Use Committee (IACUC, #A4425-01; Protocol #RCPJC1619; Date of Approval 10 May 2016).

### 4.2. Peptide and Drug

Acylated ghrelin was purchased from Sigma (St. Louis, MO, USA) while Ex-4 and Ro60-0175 were obtained from Tocris (Minneapolis, MN, USA). Ghrelin, Ex-4, and Ro60-0175 were each dissolved in sterile physiological saline prior to administration. For all central injections into the VTA, the volume delivered was 0.2 µL, whereas IP injections were administered in a volume of 1 mL/kg. 

### 4.3. Stereotaxic Surgery

Rats were anesthetized with intraperitoneal (IP) ketamine (100 mg/kg, Henry Schein, Melville, NY, USA) and IP xylazine (5 mg/kg, Sigma, St. Louis, MO, USA) and placed in a Kopf stereotaxic frame with the incisor bar set 3.5 mm below the interaural line. Animals were unilaterally cannulated with a stainless steel guide cannula (22-gauge, Plastics One), placed 4mm dorsal to the VTA (5.3 mm, ± 1.0 mm, 4.1 mm) in accordance with coordinates from [68]. Guide cannulae were secured with acrylic cement and stainless steel stylets. Stylets were changed regularly to maintain patency. After surgery, rats were single housed and given 14 days to recover before behavioral testing was initiated. Cannulae placement was confirmed using histological verification as described previously [15].

### 4.4. Design and Procedure

All rats were first trained on a progressive ratio 3 (PR3) reinforcement schedule with banana flavored sucrose pellet reinforcers (Product# F0024, Bio-Serve, Noyes, Lancaster, NH, USA). In order to establish consistent responding, training sessions were conducted daily for two weeks with each rat tested over a 2 h period at the beginning of the nocturnal cycle. Methodological details related to training, including manual shaping, have been documented previously [43]. Specifically, during training rats were initially exposed to a single fixed interval (FI) session, with a sucrose pellet dispensed every 30 s. After one session of FI training, all rats were then manually shaped to respond to the fixed ratio 1 (FR1), where each response was rewarded with a sucrose pellet. After animals responded consistently to an FR1, the schedule of reinforcement was gradually increased to fixed ratio 10 (FR10) over three sessions (further details are provided in Figure S1). It was at this point that rats were placed on the PR3 schedule until they demonstrated stabilized performance. For each experiment a repeated measures design was used with rats subjected to each treatment condition administered in a randomized order. At least four non-injection days separated successive test sessions. 

In the present study, an initial group of rats was administered ghrelin into the VTA at doses of 3 to 300 pmol in order to assess the effectiveness of each dose of ghrelin in producing an increase in operant responding. Next, to examine a possible interaction between ghrelin and GLP-1 signaling, a separate group of rats received varying doses of Ex-4 (IP, 0.1–1.0 µg/kg; VTA, 0.01–0.1 µg) co-administered with 300 pmol of ghrelin or saline vehicle injected into the VTA. Finally, in a third group of rats, IP (0.25–1.0 mg/kg) and VTA (1.5–3.0 µg) Ro60-0175 was co-administered with 300 pmol ghrelin or saline vehicle into the VTA. The doses of ghrelin, Ex-4, and Ro60-0175 are based on prior work investigating operant and appetitive responding in rodents [2,6,22,24]. The test session for each experiment was 30 min and during this time the number of reinforcers obtained was measured. Data were analyzed by separate one and two way repeated measures analysis of variance followed by post-hoc Tukey tests where justified.

## Supplementary Materials

Supplementary materials can be found at http://www.mdpi.com/1422-0067/20/4/889/s1, Figure S1: Timeline of operant training.
