# Supplementary material for: Glucagon-Like Peptide-1 (GLP-1) and 5-Hydroxytryptamine 2c (5-HT2c) Receptor Agonists in the Ventral Tegmental Area (VTA) Inhibit Ghrelin-Stimulated Appetitive Reward
Source: Int J Mol Sci. 2019 Feb 19;20(4):889. doi: 10.3390/ijms20040889 (PMC6412472; doi:10.3390/ijms20040889)
Supplement: Supplementary file 1 [file ijms-20-00889-s001.pdf]

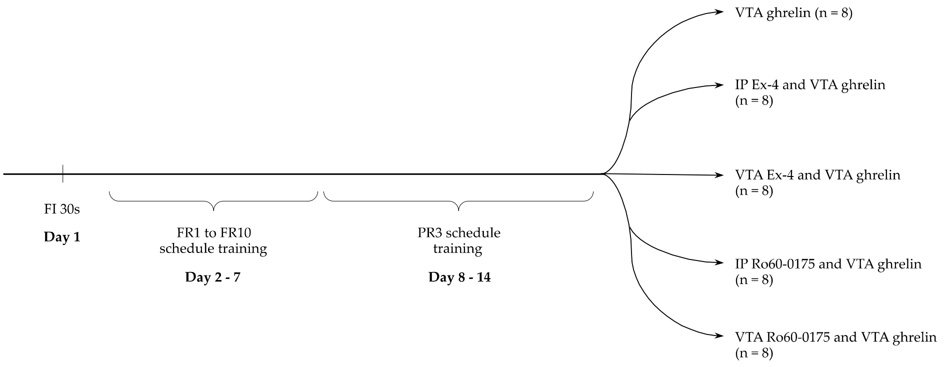

Supplemental Figure S1. Timeline of operant training. Rats were initially placed on a 30 s fixed interval (FI 30 s) schedule for banana flavored sucrose pellet reinforcers for one day. Beginning on day 2, animals were manually shaped with a fixed ratio schedule 1 (FR1), in which each single correct response was rewarded with a sucrose pellet. Following consistent responding to the FR1, the schedule of reinforcement was gradually increased to FR 10, in which 10 correct responses was rewarded with a sucrose pellet. Fixed ratio training ended on day 7, and from day 8 through day 14, rats were trained on a progressive ratio 3 (PR3) paradigm. On the PR3, the number of lever presses to earn a food reward was increased by 3 after each reward. Subsequently rats were separated into groups for intraperitoneal (IP) or ventral tegmental area (VTA) administration of ghrelin, exendin-4 (Ex-4), and Ro60-0175.
